# Degraded Land Restoration in Reinstating CH_4_ Sink

**DOI:** 10.3389/fmicb.2016.00923

**Published:** 2016-06-14

**Authors:** Jay Shankar Singh, Vijai K. Gupta

**Affiliations:** ^1^Department of Environmental Microbiology, Babasaheb Bhimrao Ambedkar UniversityLucknow, India; ^2^Molecular Glyco-biotechnology Group, Discipline of Biochemistry, School of Natural Sciences, National University of Ireland GalwayGalway, Ireland

**Keywords:** agriculture, degraded lands, forests, GHGs, methanotrophs

## Abstract

Methane (CH_4_), a potent greenhouse gas, contributes about one third to the global green house gas emissions. CH_4_-assimilating microbes (mostly methanotrophs) in upland soils play very crucial role in mitigating the CH_4_ release into the atmosphere. Agricultural, environmental, and climatic shifts can alter CH_4_ sink profiles of soils, likely through shifts in CH_4_-assimilating microbial community structure and function. Landuse change, as forest and grassland ecosystems altered to agro-ecosystems, has already attenuated the soil CH_4_ sink potential, and are expected to be continued in the future. We hypothesized that variations in CH_4_ uptake rates in soils under different landuse practices could be an indicative of alterations in the abundance and/or type of methanotrophic communities in such soils. However, only a few studies have addressed to number and methanotrophs diversity and their correlation with the CH_4_ sink potential in soils of rehabilitated/restored lands. We focus on landuse practices that can potentially mitigate CH_4_ gas emissions, the most prominent of which are improved cropland, grazing land management, use of bio-fertilizers, and restoration of degraded lands. In this perspective paper, it is proposed that restoration of degraded lands can contribute considerably to improved soil CH_4_ sink strength by retrieving/conserving abundance and assortment of efficient methanotrophic communities. We believe that this report can assist in identifying future experimental directions to the relationships between landuse changes, methane-assimilating microbial communities and soil CH_4_ sinks. The exploitation of microbial communities other than methanotrophs can contribute significantly to the global CH_4_ sink potential and can add value in mitigating the CH_4_ problems.

## An Overview of Current Atmospheric CH_4_ Status

Methane (CH_4_) is one of the most important greenhouse gases, accounting for ∼ 15–25% of the global warming (Zhou et al., 2013). CH_4_ sources are variable but their figure and enormity appear to be on the increase, while CH_4_ sinks are more unpredictable (Aronson et al., 2013). The chief global CH_4_ sources are natural and flooded soils, which contribute around one third of annual emissions (IPCC, 2007). Anthropogenic sources, including paddy agriculture, domestic cattle, landfills, fossil fuel burning, as well as biomass use for energy and agriculture, has been estimated to be >60% of total emissions (Wang et al., 2004). Moreover, it seems that the overall contribution of CH_4_ to the global emissions has been underestimated, since some studies demonstrated that CH_4_ can be readily formed *in situ* by terrestrial plants (Keppler et al., 2006). This newly identified source may have important implications in the global CH_4_ budget, and may call for a reconsideration of the role of natural CH_4_ sources in the ongoing climate change. These new findings are likely to increase the importance of CH_4_ sinks in the mitigation of its atmospheric concentrations.

**FIGURE 1:**
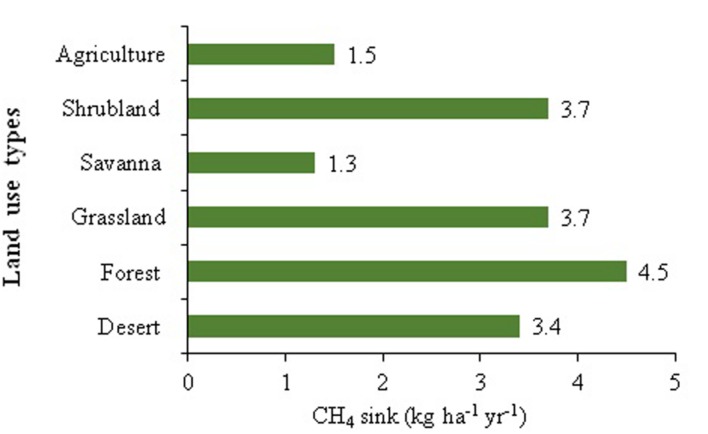
**Methane sink activity across different land uses (Developed from Levine et al., 2011)**.

It is well accepted that anthropogenic activities (landuse changes and use of chemicals in agriculture) are contributing to the global declining soil CH_4_ sink potential (Zheng et al., 2010). Landuse type is one of the major causes of soil characteristics variations and consequently the CH_4_ sink activity. It may be argued that a higher rate of CH_4_ consumption in forest soils, compared to other ecosystems could be attributed to the greater viable population size of methanotrophs (**Figure 1**). However, the experimental evidences for such arguments are still to be investigated. During the last few decades, the atmospheric concentration of CH_4_ has increased dramatically because of the imbalance between the overall sources and sinks (Singh, 2011). Anthropogenic nitrogen enrichment, grazing, deforestation, and alterations in water availability and temperature have received particular attention in terms of their effects on soil CH_4_ oxidation strength (Smith et al., 2000; Dai et al., 2013). The recent reports indicate that the problems of excess atmospheric load of this potent GHG can be mitigated either by reducing CH_4_ emissions or enhancing its consumption (Singh, 2011). It may be argued that ∼10% uplifting in the soil CH_4_ consumption could stabilize the current problem of atmospheric CH_4_ buildup (Singh, 2011). Consequently, it is imperative to adopt a more viable and eco-friendly approaches that could significantly contribute to augmentation of soil CH_4_ sink potential.

In environments the high CH_4_ content (freshwater wetlands, rice paddies, landfills, etc.) release to the atmosphere is controlled by methanotrophs via “low-capacity oxidation”. According to current concepts, the phylogenetically diverse uncultivable “high-affinity methanotrophs” are dominants in undisturbed soils and believed to be responsible for significant amount of atmospheric CH_4_ destruction. Landuse changes (conversion of natural forest and grassland ecosystems to agricultural lands) are the major consequences to the reduced soil CH_4_ oxidation rate, and are likely to persist more extensive due to anthropogenic activities. Even when restored back to its original form, previously cultivated land may have continuous lower CH_4_ sink activity than undisturbed lands. Variations in CH_4_ uptake strength in soils under different landuse practices could be due to the variation in methanotrophic community types. Based on the above arguments, it may be hypothesized that restoration of soil CH_4_ sink strength in restored degraded land could be correlated to the variation in restored methanotrophic bacterial community composition. However, only a few studies addressed to methanotrophic activity and diversity in restored lands (Knief et al., 2005; Dorr et al., 2010; Levine et al., 2011; Zhou et al., 2013). The methanotrophs distribution and their CH_4_ sink potential under prevailing influential environmental factors in resorted soils are still enigmatic. In addition, investigations on the influence of plant species on restored soil methanotrophs distribution are still very limited (Degelmann et al., 2010; Dorr et al., 2010), and the variation of methanotrophic communities in rehabilitated land with different plantation ways (natural and managed) remains unresolved (Dai et al., 2015).

The previous investigations have typically assessed either CH_4_ flux or the microbial methanotrophic community at similar ecosystems, but hardly ever has the role mediated by the efficient methanotrophic population size/community been studied concomitantly across a land-use gradient. My proposal is not to untangle the origin causes of difference in either methanotrophic community structure or CH_4_ flux, but to explain a relationship that, if directional, possibly will suggest insights into long-term management strategies for the methanotrophic community and CH_4_ flux. To our knowledge, there is no study to correlate methanotroph and bacterial diversity to both the stability and magnitude of the associated *in situ* CH_4_ sink across a landuse gradient, and to exploit the results to put on insights into management practices for abatement of atmospheric CH_4_ gas increases. Therefore, there is urgent need to investigate the methanotrophs abundance and diversity variations correlated with the atmospheric CH_4_ sink potential in restored soils under varied environmental regimes and dominant/selected vegetation cover.

## Methane-Assimilating Mircobes and CH_4_ Sink

Among the microbes, CH_4_-consuming bacteria (primarily methanotrophs), ammonium-oxidizers (a type of nitrifiers) and sulfate-reducing bacteria (SRBs) are the key microbial groups that consume CH_4_ (Strong et al., 2015). SRBs reduce sulfate into sulfide using CH_4_ as a source of energy (Knittel and Boetius, 2009). *Verrucomicrobia*, a recently discovered group of CH_4_-utilizing microbes that consists of thermophiles, has also been identified to the group of known CH_4_-assimilating bacteria (Kalyuzhnaya et al., 2015). In addition to the more common methanotrophs, a group of anaerobic CH_4_-oxidizing *Archaea* has been also reported to be involved in denitrification coupled with anaerobic CH_4_ oxidation (Raghoebarsing et al., 2006). However, it seems that microbes such as nitrifiers, SRBs, *Archaea*, etc. involved in CH_4_ assimilation other than methanotrophs cannot grow as usual as aerobic CH_4_-oxidizing methanotrophs (Jones and Morita, 1983). In hypothesis, microbes should be capable of using inorganic nitrogen to assimilate CH_4_ anaerobically, but such microorganisms have neither been assessed in nature nor isolated in the laboratory (Raghoebarsing et al., 2006). Therefore, the potential contribution of such microbes in global CH_4_ sink phenomenon is largely unexplored and needs urgent attention (Singh, 2015).

**FIGURE 2:**
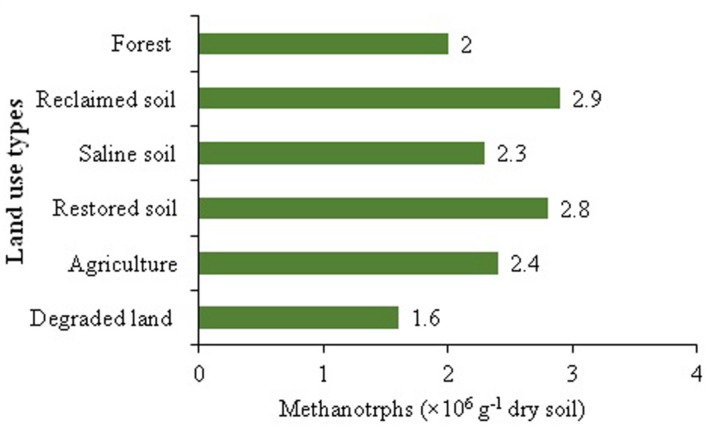
**Methanotrophs abundance across different land uses (Developed from Tiwari et al., 2015)**.

Due to the key role of methanotrophs in the biogeochemical carbon cycle and in global climate change, the influence of landuse on methanotrophs diversity has attracted ample consideration (Robertson et al., 2000). A number of studies have been performed to evaluate the influence of different landuse changes on the CH_4_ uptake capacity with contrasting results (Prieme and Christensen, 1999; Suwanwaree and Robertson, 2005; Singh et al., 2008). For instance, Menyailo et al. (2008) confirmed that landuse patterns suppressed the soil CH_4_ uptake without affecting the diversity of methanotrophs. Similarly, tree species affected atmospheric CH_4_ oxidation in grassland soil exclusive of altering methanotrophic community (Menyailo et al., 2010). The lower population size of methanotrophs in degraded land soil could be due to the disturbances in soil conditions and micro-ecological niches of the bacteria (**Figure 2**). It may be argued that variations in CH_4_ oxidation and methanotrophs diversity and abundance may largely result from, and soils analyzed across a wide variety of habitats, however, the exact explanations about these arguments have not been fully addressed.

Methanotrophs are the only known potential biological sink for CH_4_ in terrestrial/upland soils (Singh, 2011; Pandey et al., 2014). It has been suggested that deforestation and land management practices alter the soil characteristics, which in turn, adversely affect the viable soil methanotrophic community and their CH_4_ sink activity (King and Nanba, 2008; Dorr et al., 2010). However, reforestation may be the promising strategy to promote terrestrial CH_4_ uptake by soils owing to recovery of methanotrophs diversity. The afforestation of pastures experienced a shift in the methanotrophs community in soils from three different rainforest sites (Singh et al., 2007). Similarly, the conversion of natural forest to agricultural land also lowered the CH_4_ consumption by soils; nevertheless, it could be restored through revegetation (Livesley et al., 2009; Zhang et al., 2014). A recent field study from natural ecosystem suggests that soil restoration, even if performed within a rather limited time, may have the positive effect on CH_4_ consumption by terrestrial soils (Kizilova et al., 2013). It is suggested that the bio-fertilizer can be applied to minimize CH_4_ emission from flooded paddy soils and also holds promise as the efficient device for controlling the potent greenhouse gas CH_4_.

Many studies claimed that long-term N fertilization in paddy soils alters the methanotrophic composition, resulting in inhibited CH_4_ oxidation (Bodelier et al., 2000; Bodelier and Laanbroek, 2004; Mohanty et al., 2006; Noll et al., 2008; Banger et al., 2012; Zheng et al., 2013). Several management practices, including the organic amendments and residue managements have been proposed to restore the microbial diversity and methanotrophs numbers in deteriorated agricultural lands (Singh et al., 2011; Singh and Pandey, 2013; Carlson et al., 2015). It has been proposed that direct introduction of beneficial bio-filmed bio-fertilizers may significantly contribute to the revival of microbial diversity in degraded agro-ecosystems (Singh et al., 2011). Bio-fertilizer applications, particularly the nitrogen-fixing bio-agents, such as cyanobacteria, free-living diazotrophs, and *Azolla* may augment methanotrophs diversity and CH_4_ oxidation while reducing the amount of N fertilizer applied (Singh and Strong, 2016; Singh et al., 2016). Cyanobacteria are the exceptional model systems that can offer the biotechnologist with novel genes and stress tolerance capability having diverse uses in environmental sustainability and also; hold promise as the effective tool for harnessing CH_4_ (Prasanna et al., 2002). It may be argued that applications of above-described microbial bio-fertilizers in place of their chemical counterparts may mitigate the onset of global CH_4_ emission by conserving the viable methanotrophs diversity in disturbed agriculture soils (Pingak et al., 2014). The application of these bio-agents in paddy fields may be considered as the innovative tool for promoting the methanotrophs community composition. It is suggested that the application of bio-fertilizers as the nitrogen fertilizer replacement would be cost-effective, eco-friendly, and the safer means for degraded land restoration, and also to conserve the methanotrophic diversity and CH_4_ consumption in the long term.

## Conclusions and Future Prospects

It is comprehensible from the above findings that ecological distribution, diversity, and CH_4_ sink potential of methanotrophs are largely affected by landuse changes. However, pertaining to the growth and activity of the methanotrophic community with CH_4_ sinks and the physico-chemical soil conditions remains an unresolved to understand the underlying mechanisms driving the methanotrophs–CH_4_ sink phenomenon. As postulated, a drastic change in soil chemical properties in a given ecosystem owing to landuse changes could be a potential threat to adversely affect both the CH_4_ sink activity and community composition of methanotrophs. However, in restored degraded ecosystems the improved and favorable soil conditions could help to establish and recovery of methanotrophs diversity. The use of bio-fertilizers, in place of inorganic nitrogen fertilizers, can enhance the optimum nitrogen availability to plants and CH_4_ assimilation efficiency of methanotrophs in the agriculture soils. It is possible that spore-forming methanotrophs, lying dormant in the disturbed soils owing to unfavorable environmental conditions, might be instrumental in restoring methanotrophic bacterial diversity once the ecological conditions were favorable (Jasper, 2007). The restoration of the ecological niche of methanotrophs in reforested and recovered soils would possibly favor the diverse methanotrophs community establishment, and to perform optimally (Singh and Singh, 2013). However, information on CH_4_-consuming bacterial diversity under different landuse types is still in a very incipient state. It is also not known whether different disturbed ecosystems following restoration have similar or different CH_4_-consuming bacterial community compositions. Further, there is no knowledge on viability of different methanotrophic community compositions (Type I or II) in a given re-established ecosystem. Even when rehabilitated back to its natural state, previously cultivated land continues to have a lower CH_4_ oxidation rate than undamaged areas. This apparent irreversibility of human impact on the CH_4_ oxidation rates has important implications for the future of land management strategies. It is expected that only future research investigations would possibly provide answer to some of the questions raised so far. Further, an understanding of the microbial agents other than CH_4_-consuming bacteria and its physiological behavior in the reclaimed/restored degraded soils is important to verify the significant contribution of such microbes in soil CH_4_ sink event. Therefore, the increased abundance and community composition of viable methanotrophs in soils of restored ecosystems could be the innovative approach to notably enhance the strength of CH_4_ uptake. Herein a list of few directions for future research on the ecology of methanotrophs in restored soils is anticipated:

(1) Since the soils of restored lands will have heterogeneous complex substrates and may be affected by many influential environmental factors including abnormal climatic conditions, the unique bacteria living in the soils will vary greatly by space and time. A short time-frame study on this aspect might bias our results and understanding; long-term monitoring of methanotrophs biodiversity and abundance in restored soils will enhance our understanding of their viability, stability, and CH_4_ sink potential.
(2) Would global climate change have any direct effect on abundance and methanrophs species in the soil of transformed lands? How do these soil microbes adapt or acclimate to global climate change?
(3) It assumed that in a restored or derived ecosystems the newly exotic microbial flora such as nematodes may influence the potential of CH_4_ oxidation indirectly through exerting their effect on efficiency and number/diversity of methanotrophs.

## Author Contributions

JSS contributed the about role of land restoration in retrieving soil methanotrophs diversity and methane sink potential. VKG discussed about various arguments related to the methanotrophs and their roles in methane consumption under different lands use.

## Conflict of Interest Statement

The authors declare that the research was conducted in the absence of any commercial or financial relationships that could be construed as a potential conflict of interest.
